# Carbonite

**Published:** 1874-12

**Authors:** Weston F. Milliken, W. W. Brown, James E. Carter, James S. Marrett, L. D. M. Sweat, George T. Davis, James W. Cummings

**Affiliations:** Portland; Portland; Portland; Portland; Portland; Portland; Portland


Carbonite.—Since our November issue, in which we gave our opinion of the value of this new fuel, we have received the following from prominent citizens of Portland, Maine. So far as we have information, the favor in which we hold this article is shared by all familiar with it.

Portland, Oct. 26th, 1874.

We are now burning the new fuel, called "Carbonite," owned by the James River Coal Company, and can recommend it as the most desirable fuel we have ever used in open grates, both for its cleanly and lasting qualities.

Weston F. Milliken
W. W. Brown,
James E. Carter,
James S. Marrett,
L. D. M. Sweat,
George T. Davis,
James W. Cummings.

